# Clinical Outcome and Utility of Cone‑Beam Computed Tomography Imaging for Transcatheter Arterial Embolization in Patients with Malignant Intractable Hematuria

**DOI:** 10.5334/jbsr.3781

**Published:** 2025-02-03

**Authors:** Chang Hoon Oh, Hyo Jeong Lee, Sang Lim Choi

**Affiliations:** 1 Department of Radiology, Ewha Womans Mokdong Hospital, College of Medicine, Ewha Womans University, Seoul, Republic of Korea; 2 Department of Radiology, Chung‑Ang University Gwangmyeong Hospital, Gwangmyeong, Republic of Korea

**Keywords:** Hematuria, Embolization, Bladder cancer, Cone‑beam computed tomography, Angiography

## Abstract

*Background:* We assess the clinical outcomes and utility of cone‑beam computed tomography (CBCT) during transcatheter arterial embolization (TAE) in patients with malignant intractable hematuria, related to lower urinary tract malignancy.

*Methods:* A total of 22 consecutive patients (20 males and 2 females; age 71.8 ± 9.6 years) underwent CBCT during TAE for malignant intractable hematuria from May 2023 to August 2024. CBCT was performed on both internal iliac arteries for selective imaging. Contrast‑enhanced three‑dimensional (3D) images were acquired during breath‑hold for embolization planning, with automated feeder detection aiding vessel visualization. Follow‑up CT was performed 2–3 months after TAE, and regular visits monitored hematuria recurrence and treatment effects.

*Results:* In all, 27 TAE procedures were performed in 22 patients, including those with bladder and prostate cancers. Technical success was achieved with all procedures. Clinical improvement in hematuria was observed in 86.4% of patients within 2 days. Five patients required re‑intervention, and all improved. Significant changes were noted in hemoglobin, heart rate, transfusion, and tumor size, with 85.7% showing tumor reduction on follow‑up imaging. CBCT provided valuable information in 52.1% cases, leading to treatment plan adjustments, particularly in identifying additional feeders and enabling superselective embolization. No major complications were reported.

*Conclusion:* TAE is a safe and effective treatment for malignant intractable hematuria, leading to significant clinical improvement. CBCT further enhances TAE by providing crucial imaging that optimizes the embolization process.

## Introduction

Hematuria from malignancy is a significant clinical challenge due to its life‑threatening nature and impact on patient outcomes [1]. Conservative treatments often fail to provide long‑term relief [2, 3], and while surgical hemostasis is effective, it poses high morbidity and mortality risks, particularly for the elderly or unresectable patients [4, 5]. Transcatheter arterial embolization (TAE) has become crucial for managing hematuria in bladder cancer and other malignancies, offering a minimally invasive alternative for patients in whom surgery is unsuitable or who are unresponsive to other treatments [5]. TAE has advanced, targeting vesical and prostatic arteries [6], and offers benefits such as fewer complications, lower mortality, and improved quality of life [5].

Cone‑beam computed tomography (CBCT) is useful in various interventional radiology procedures, such as transarterial chemoembolization (TACE), bronchial artery embolization (BAE), and prostatic artery embolization (PAE) [7–10]. It enhances vessel visualization and reduces non‑target embolization risk, offering advantages over digital subtraction angiography (DSA) alone [11]. Despite the apparent advantages of CBCT, the use of imaging technology during TAE for intractable hematuria has rarely been reported.

Given the lack of studies on the effectiveness of TAE and the utility of CBCT during procedures for intractable hematuria, this study aims to assess the clinical outcomes and utility of CBCT during TAE in patients with malignant intractable hematuria.

## Materials and Methods

### Patient population

**Figure 1:**
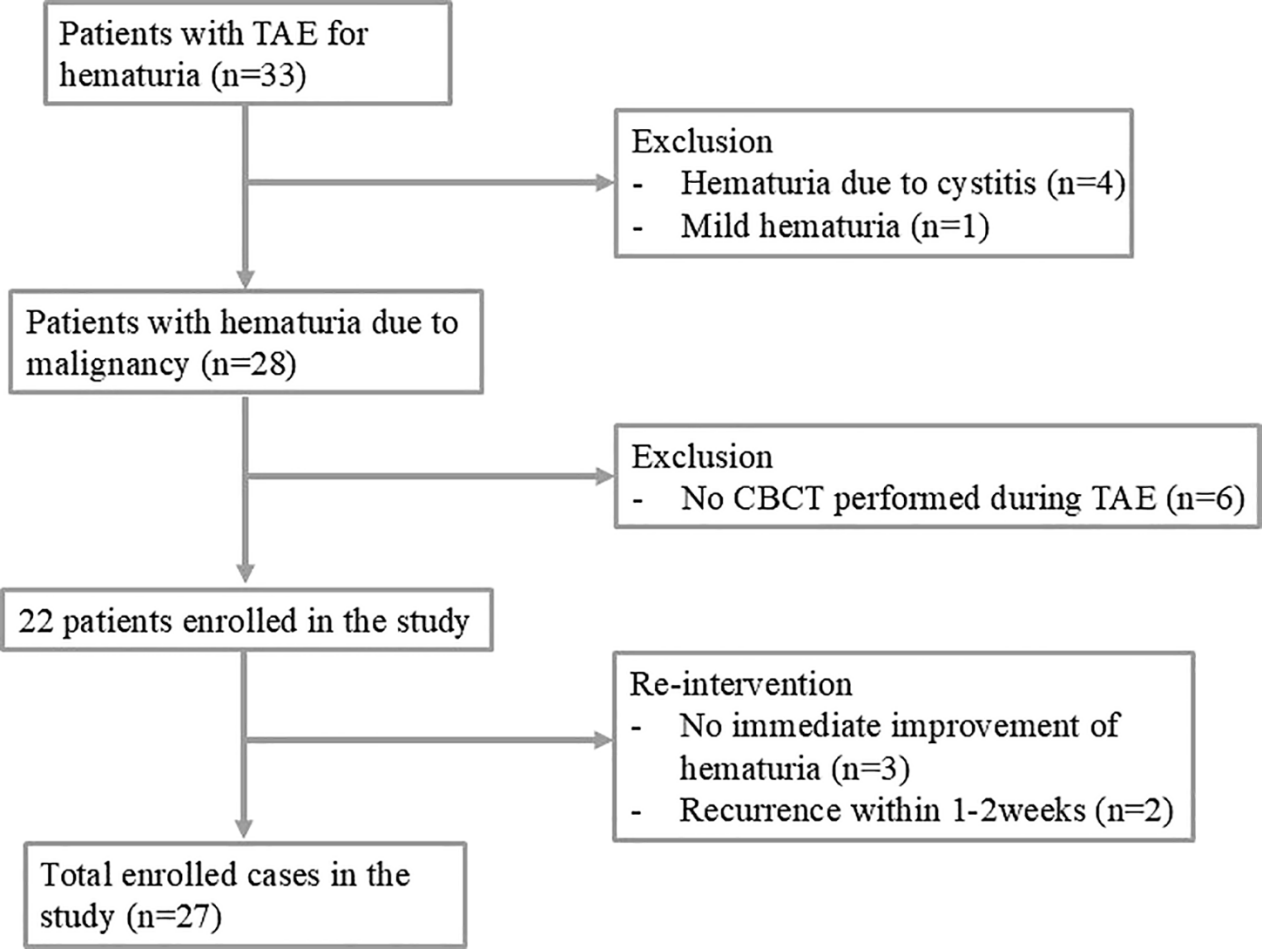
Flowchart of transcatheter arterial embolization in patients with malignant intractable hematuria.

The databases were queried from May 2023 to August 2024,and 22 patients (20 males, 2 females; age 71.8 ± 9.6; 19 bladder cancer and 3 prostate cancer) were included (Table 1). Written informed consent for the interventional procedures was obtained from all patients. Candidates for TAE were those in whom conventional treatments such as bladder irrigation with formalin or silver nitrate, intravesical hydrostatic pressure, and endoscopic diathermy had failed, or who required transfusions due to low hemoglobin. All patients had gross hematuria from malignancies (bladder or prostate cancer) requiring continuous bladder irrigation (CBI) with Foley catheter insertion. Seven patients underwent radical cystectomy shortly after TAE. These patients had already been scheduled for surgery but required TAE due to severe hematuria that persisted despite conservative treatment. Patients with non‑malignant causes or mild hematuria (scale score: 1 or 2) were excluded (Figure 1).

**Table 1:** Baseline characteristics of the study population (*n* = 22).

CHARACTERISTICS	VALUE
Age	71.8 ± 9.6
Sex Male Female	20 (90.9%) 2 (9.1%)
Malignancy Bladder cancer Prostate cancer	19 (86.4%) 3 (13.6%)
Number of TAE 1 2	17 (77.3) 5 (22.7)
Follow‑up (months)	7.6 ± 4.8

TAE, transcatheter arterial embolization.

### Procedure

The right common femoral artery was accessed under local anesthesia, and a 5‑F Cobra catheter (Cook, Bloomington, IN, USA) was advanced for selective contralateral internal iliac artery (IIA) catheterization. The ipsilateral IIA was selected using the same Cobra catheter after forming a Waltman loop. A 5‑F catheter was placed bilaterally in the IIAs for CBCT imaging. Based on angiographic and CBCT findings, superselective catheterization of the feeding arteries to the bladder was performed using a 1.7–1.9 Fr coaxial microcatheter (Veloute or Tellus; ASHAI INTECC, Aichi, Japan).

**Figure 2:**
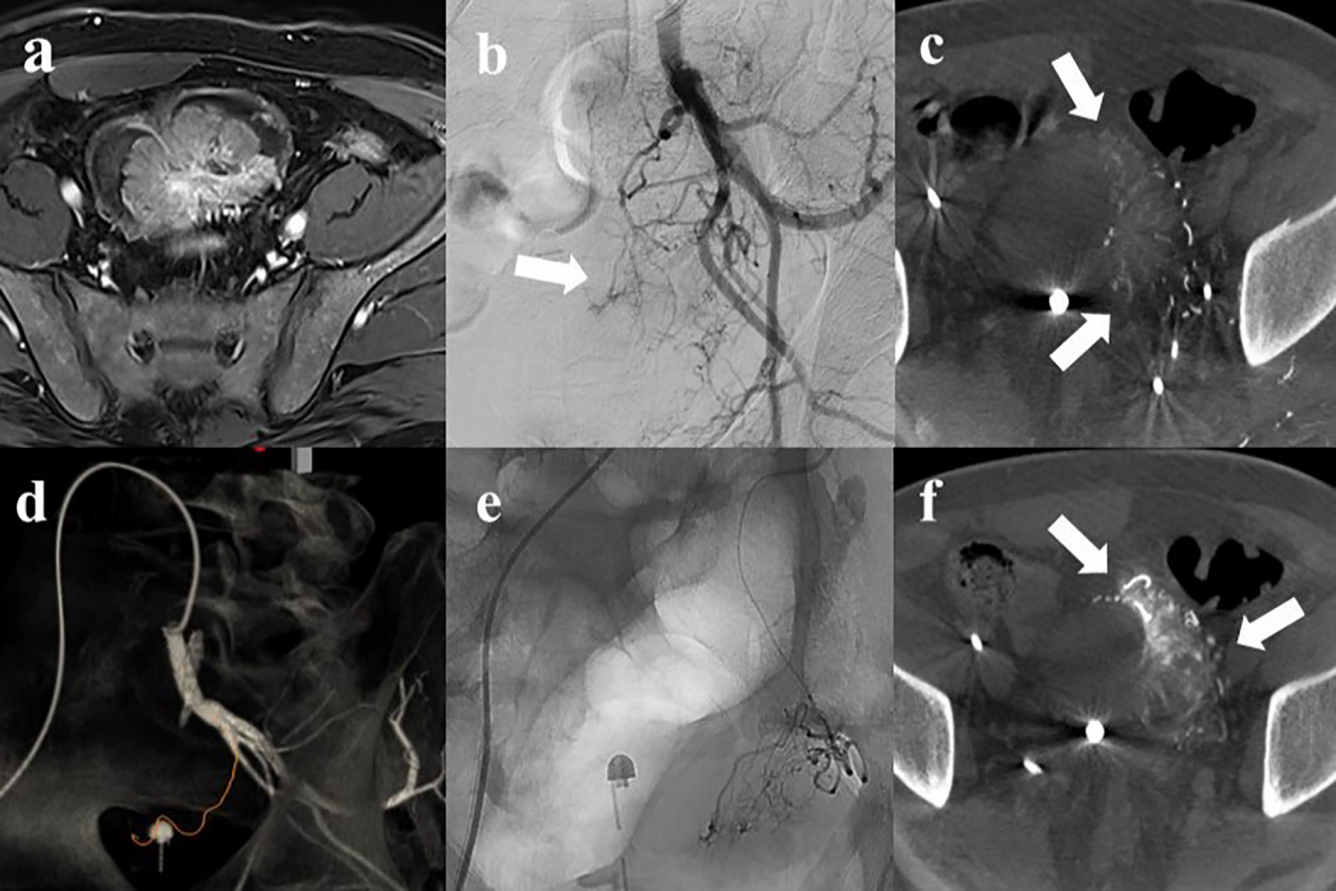
A 53‑year‑old male with intractable hematuria due to bladder cancer. (**a**) Magnetic resonance imaging revealed a 7 cm enhancing bladder dome mass. (**b**) Left internal iliac angiography showed hypervascular tumor staining (arrow) with complex vascular anatomy. (**c**) Axial cone‑beam CT (CBCT) showed perfusion to the left side of the tumor (arrow). (**d**) Automated feeder detection identified the vessel pathway (red line) leading to the tumor. (**e**) Superselective embolization was performed using tris‑acryl gelatin microspheres. (**f**) Post‑embolization CBCT revealed contrast retention (arrow) within the tumor, followed by contralateral embolization using the same protocol.

CBCT was performed on both IIA. A microcatheter was used for selective CBCT when needed. A total of 15–20 mL 50% diluted contrast medium was injected automatically at 1.5–2 mL/s according to the vessel diameter with a 4‑s imaging delay. CBCT images were obtained during a breath‑hold, with a 6‑s acquisition time covering 220 rotations. The acquired images were automatically transferred to three‑dimensional (3D) workstations (Leonardo Syngo X; Siemens Medical Systems, Munich, Germany). Embolization planning was supported by automated feeder detection (AFD) software (Syngo Embolization Guidance; Siemens Medical Systems). After manually pointing to the vessel of interest and following it, the entire path of the target vessel was rendered as a 3D roadmap (Figure 2).

Embolic agents included 100–300 μm tris‑acryl gelatin microspheres (Embosphere®; Merit Medical Systems, South Jordan, UT, USA), a 1:2 n‑butyl cyanoacrylate (Histoacryl; B. Braun, Melsungen, Germany)/iodized oil (Lipiodol, Guerbet, Roissy, France) ratio, detachable vascular microcoils (Concerto; Medtronic, Minneapolis, MN, USA), and 100–300 μm gelatin sponge particles (Nexsphere; Next Biomedical, Incheon, South Korea) or their combinations. Embolic materials were selected intraoperatively on a case‑by‑case basis, at the discretion of the interventional radiologists (H.J.L. and C.H.O.).

### Follow‑Up and study endpoints

Follow‑up computed tomography (CT) was performed 2–3 months after TAE. Additionally, the next steps in treatment were determined through regular follow‑up visits to monitor the recurrence of hematuria and evaluate the therapeutic effect. Technical success was defined as targeted vessel occlusion on post‑embolization arteriography. The significant reduction in tumor size was defined as a ≥30% decrease in the sum of the longest diameters of target baseline lesions on follow‑up imaging (or via enhancement imaging during the arterial phase). Recurrence of bleeding was defined as the recurrence of hematuria requiring additional treatment after successful cessation of bleeding. The severity of hematuria was assessed on the basis of the gross hematuria scale in patients who had undergone CBI [12]. Complications were classified into minor and major categories according to the Society of Interventional Radiology criteria [13].

### Data analysis

Patient imaging files were archived in the institutional picture archiving and communication system (PACS) system and retrospectively reviewed, along with procedure reports, by two board‑certified interventional radiologists specializing in vascular interventions. CBCT findings classified each case into one of three categories (Table 2). Discrepancies were resolved through collaborative review. Intraoperative images were evaluated to identify potential non‑target embolization sites, additional tumor‑feeding arteries, and contrast retention within the tumor or unintended areas post‑procedure.

**Table 2:** Interpretive categories and criteria for CBCT for embolization in patients with intractable hematuria.

Category 1	CBCT provided no additional diagnostic information. Added no unique information beyond the DSA findings.
Category 2	CBCT supplied supplementary information without necessitating a change in the therapeutic approach. Clarified questionable findings observed on DSA. Established a correlation between vascular territory and the target lesion.
Category 3	CBCT yielded pertinent findings that prompted a revision of the treatment strategy. Led to the embolization of an additional tumor‑feeding artery. Led to the more superselective catheterization of the target vessel due to non‑target embolization through the vascular anastomosis.

CBCT, cone‑beam computed tomography; DSA, digital subtraction angiography.

## Results

Five patients (three with bladder cancer, two with prostate cancer) required re‑intervention, leading to 27 total procedures (Table 3). Hematuria improved within 2 days for 19 of 22 patients (86.4%). Technical success was achieved in all procedures. For prostate cancer patients, nine side prostatic arteries were embolized (four bilateral, one unilateral). In bladder cancer cases, 22 procedures targeted vesical arteries, with 17 bilateral and 5 unilateral embolizations, occluding 61 vessels. Superselection failed in two arteries due to tortuosity, requiring microcoil placement at the distal IIA and gel foam embolization of the vesical artery.

**Table 3:** Summary of characteristic details of total 27 procedure cases.

CHARACTERISTICS	VALUE
Time to selection of target vessel	9.6 ± 7.1 min
Fluoroscopic time	23.5 ± 8.2 (11.0 ~ 45.0) min
Number of CBCT scans	2.82 ± 1.05
Bilaterality Unilateral Bilateral	6 (22.2%) 21 (77.8%)
Embolic materials TAGM GSP NBCA with iodized oil Microcoils Multiple agents	26 (96.3%) 12 (44.4%) 2 (7.4%) 2 (7.4%) 11 (40.7%)
Embolized arteries Superior vesical artery Inferior vesical artery Prostatic artery Other internal iliac branches	49 36 9 2
Category 1 2 3	4 (8.3%) 19 (39.6%) 25 (52.1%)

CBCT, cone‑beam computed tomography; TAGM, tris‑acryl gelatin microspheres; GSP, gelatin sponge particles; NBCA, n‑butyl cyanoacrylate.

Three patients had initial failure but responded to re‑intervention within 1–2 weeks. Specifically, three patients (two with TAGM and GSP combined, one with only GSP) experienced initial failure despite achieving hemostasis at the end of the first embolization session. They showed target‑vessel recanalization on follow‑up angiography within 1–2 weeks and improved after repeat superselective embolization. Two patients experienced recurrence after a month and required additional TAE; all improved post‑re‑intervention.

Table 4 presents the comparison of laboratory data and tumor size before and after the procedure. Hemoglobin, heart rate, transfusion, and tumor size demonstrated statistically significance. Follow‑up imaging revealed significant tumor reduction in 12 out of 14 patients (85.7%). In patients who underwent radical cystectomy, the time interval between TAE and cystectomy was 7.86 ± 2.36 days.

**Table 4:** Comparison of laboratory data and tumor size before and after the procedure.

CHARACTERISTICS	PRE‑PROCEDURE	POST‑PROCEDURE	*P*‑VALUE
Hemoglobin (g/dL)	7.48 ± 1.73	8.86 ± 1.69	<0.001
Blood pressure (mm Hg)	126.6 ± 11.6	132.9 ± 12.6	0.080
Heart rate (per min)	83.5 ± 16.7	76.7 ± 10.1	0.009
Platelet count	214.5 ± 97.3	206.6 ± 99.7	0.571
INR	1.10 ± 0.09	1.08 ± 0.84	0.105
Transfusion (pRBC)	2.41 ± 1.68	0.64 ± 0.58	<0.001
Tumor size (cm)*	5.47 ± 1.53	3.61 ± 1.35	<0.001

INR, International nomalized ratio; pRBC, packed red blood.

*Patients who did not receive radical cystectomy after the procedure.

CBCT did not provide additional information in 8.3% of cases (4/48, Category 1). It confirmed the angiographic findings in 39.6% of cases (19/48, Category 2). For 52.1% (25/48, Category 3), CBCT altered the treatment plan, identifying complex anatomy, mismatches between vascular territories, alternative (*n* = 6) and additional feeders (*n* = 16), or non‑target embolization requiring further superselective catheterization (*n* = 3).

No major complications or changes in bladder/sexual function occurred. Fever and pain were reported in four patients (29.6%) but resolved within 2–3 days with conservative treatment.

## Discussion

Unlike traditional DSA, CBCT allows visualization in multiple planes, correlating vessels with adjacent soft tissue and bone structures when angiography lacks detail [14]. Though CBCT increases contrast media volume and procedure time, its ability to distribute radiation across a 180° arc reduces localized exposure compared with repeated DSA [14–16]. Enhanced visualization is particularly beneficial in complex pelvic anatomy, aiding complete embolization [17].

In our study, CBCT was essential for confirming or modifying the treatment plan in 91.7% of cases. Among these, 52.1% of cases (Category 3) required adjustments to the treatment plan based on additional insights provided by CBCT, such as the detection of additional feeders not clearly identifiable on DSA. This allows for precise embolization and prevents incomplete embolization. Residual blood flow to the tumor can lead to regrowth and higher recurrence rates, as incomplete embolization is significantly linked to poor control over the target lesion’s blood supply [18]. Post‑embolization CBCT can be used to check for contrast retention in the tumor, thereby confirming effective embolization. If incomplete embolization is detected, the previous CBCT can be reviewed to identify any additional feeders, offering a clear advantage for optimizing the treatment.

Furthermore, CBCT played a pivotal role in 18.8% cases in which alternative feeders were identified, and superselective catheterization was required to avoid non‑target embolization. This is particularly important when branches to critical structures such as the rectum or seminal vesicles are involved, as embolization can cause severe complications, including ischemia or necrosis, potentially necessitating surgical intervention [17]. DSA alone can struggle to distinguish between bladder tumor staining and prostate contrast blush, complicating target‑vessel identification. In addition, prostate and seminal vesicles often receive blood supply from the inferior vesical artery, which may share a trunk with other arteries or originate independently [1]. If CBCT identifies vascular supply to other organs or alternative vessels, using a superselective embolization technique helps prevent non‑target embolization by clearly distinguishing the tumor from surrounding structures, reducing misinterpretation and enhancing procedural precision [19].

Our study demonstrated that TAE was highly effective in reducing transfusion requirement (73.4%) and improving CBI outcomes (50.0%) in patients with malignant hematuria. Reducing transfusion volume is particularly beneficial in oncological patients because it minimizes blood‑transfusion‑associated risks, such as immunosuppression and transfusion‑related complications [20]. Moreover, TAE plays a crucial role in reducing CBI, which is significant in managing hematuria conservatively and reducing patient discomfort. By successfully controlling the bleeding source through targeted embolization, TAE not only improves immediate hemostasis but also decreases the need for prolonged irrigation, leading to short hospital stays and few complications. These findings align with the results of previous studies reporting that preoperative embolization reduces intraoperative blood loss and transfusion needs, particularly in highly vascular tumors [20].

In addition to controlling hematuria, TAE demonstrated tumor reduction in our study. Among the 14 patients who underwent follow‑up imaging, 12 (85.7%) exhibited significant tumor reduction with decreased enhancement on CT or magnetic resonance imaging (MRI). This suggests that TAE, while primarily aimed at controlling hemorrhage, also offers a secondary benefit in reducing the tumor burden. Furthermore, in a recent study, using drug‑eluting beads (DEBs) showed promising results in achieving both hemostasis and tumor control in patients with bladder cancer and hematuria [21]. In patients with bladder cancer and hemorrhage, DEB‑TACE provides a total effective rate (complete response [CR] + partial response [PR]) of 64.1%, with a disease benefit rate of 79.5%, making it a viable alternative for patients who cannot undergo surgery.

This study had several limitations. As a retrospective, nonrandomized study, variability in patient condition, tumor characteristics, and angiographic findings may have introduced confounding factors. The choice of target vessels and embolic agents varied on the basis of operator preference, possibly affecting outcomes. The small sample size and focus on severe hematuria may have influenced the clinical success rates. The presence of ischemic changes could not be determined from the pathologic specimens or surgical findings in patients who underwent cystectomy. Additionally, some patients underwent radical cystectomy shortly after TAE, complicating long‑term outcome assessment. The lack of a control group and risk factor analysis further limits the study. Future research with larger cohorts is needed to validate these findings.

In conclusion, TAE is a safe and effective treatment for intractable malignant hematuria and contributes to significant clinical improvement. Moreover, CBCT is a valuable tool during TAE, enhancing the decision‑making process by providing supplementary imaging information that aids in optimizing the embolization procedure for malignant intractable hematuria.
